# Case Report: Guillain–Barré syndrome with three episodes and literature review

**DOI:** 10.3389/fimmu.2025.1559937

**Published:** 2025-05-02

**Authors:** Ying Wang, Chunmei Zhao, Rong Chen, Zeli Ma, Nan Tian, Xuan Li, Xianrui Xu, Qiang Liu, Yajun Li, Fenkui She, Fenglin Mu, Qing Zhang

**Affiliations:** ^1^ Clinical Medical College, Ningxia Medical University, Yinchuan, Ningxia, China; ^2^ Department of Neurology, General Hospital of Ningxia Medical University, Yinchuan, Ningxia, China; ^3^ Department of Neuroelectrophysiology, Cardiovascular and Cerebrovascular Disease Hospital Branch, General Hospital of Ningxia Medical University, Yinchuan, Ningxia, China; ^4^ Department of Neurology, General Hospital of Ningxia Medical University, Ningxia Key Laboratory of Cerebrocranial Diseases, Incubation Base of National Key Laboratory, Yinchuan, Ningxia, China

**Keywords:** recurrent Guillain-Barré syndrome, Guillain-Barré syndrome, autoimmune diseases, peripheral neuropathy, case report

## Abstract

Guillain–Barré syndrome (GBS) is an autoimmune-mediated peripheral neuropathy that is usually monophasic, but recurrence occurs in 2%–5% of cases, termed recurrent Guillain–Barré syndrome (RGBS). We report the case of a 49-year-old male patient who experienced three episodes of bilateral lower extremity numbness and weakness, each progressing to its nadir within 4 weeks. The first episode was additionally characterized by bilateral upper extremity weakness and dyspnea, while the first two episodes were accompanied by numbness in the fingertips of both hands. Electromyography (EMG) revealed severe axonal damage with concomitant demyelination. Anti-sulfatide IgG antibodies were detected during the third episode. After excluding other demyelinating disorders, a definitive diagnosis of GBS was established, and the symptoms were nearly completely resolved following treatment with intravenous immunoglobulin (IVIG).

## 1 Introduction

Guillain–Barré syndrome (GBS) is an immune-mediated acute inflammatory peripheral neuropathy. The diagnosis of GBS is established based on the following criteria: acute onset with symptoms peaking within 4 weeks, followed by stabilization or improvement, and clinical features consistent with either classic or variant forms of GBS. Supporting evidence includes a history of antecedent factors (e.g., infection or vaccination) within 6 weeks prior to disease onset, albuminocytologic dissociation in cerebrospinal fluid (CSF), electromyography (EMG) confirmation of peripheral nerve involvement, and positive serum anti-ganglioside antibodies. The diagnosis also requires the exclusion of other etiologies of acute peripheral neuropathy (1). The diagnostic criteria for RGBS include at least two episodes of GBS, each meeting the National Institute of Neurological Disorders and Stroke (NINDS) criteria for GBS. The minimum interval between recurrence is 2 months if symptoms fully resolve or 4 months if symptoms partially resolve (2).

Differential diagnoses typically include related conditions such as Guillain–Barré syndrome with treatment-related fluctuations (GBS-TRF) and acute-onset chronic inflammatory demyelinating polyneuropathy (A-CIDP) (2). GBS-TRF is defined as a condition in which patients receiving immunotherapy exhibit initial improvement or stabilization followed by ≤2 recurrences or deteriorations within 2 months. These recurrences are characterized by at least a one-grade deterioration on the GBS Disability Scale or a reduction of ≥5 points in the overall Medical Research Council (MRC) score (3, 4). A-CIDP presents with acute symptoms similar to GBS but evolves into a chronic or relapsing course. Patients typically reach the disease nadir within 8 weeks and are generally older at onset. Clinical features of A-CIDP include prominent sensory abnormalities, vibratory hypoesthesia, and sensory ataxia, while loss of pinprick sensation, cranial nerve involvement, and autonomic dysfunction are rare. Differentiating between A-CIDP and GBS remains challenging electrophysiologically (3). Given the distinct treatment approaches for these related diseases, establishing an accurate diagnosis during the early stages of the condition is of paramount importance. We present a case of GBS characterized by three recurrent episodes, during which anti-sulfatide antibodies were detected, and explore the potential underlying causes of recurrence.

## 2 Case presentation

The patient was a 49-year-old male hospitalized on the following three occasions: 13 June 2018, 24 March 2023, and 29 August 2024, with intervals of 5 years and 1 year, respectively. All three episodes were characterized by numbness and weakness in both lower extremities, with the first two episodes also accompanied by numbness in the fingertips of both hands. The first episode was most severe, also presenting with reduced vocal volume, difficulty in eye closure, and respiratory distress. A history of upper respiratory tract infection preceded the first two episodes, while no clear antecedent infection was identified before the third episode (**Table 1**). Neurological examination revealed varying degrees of decreased muscle strength in the extremities, reduced or absent tendon reflexes, and sensory abnormalities (hypoesthesia or hypersensitivity) in all three episodes. Additionally, the first episode was marked by weakness in eye closure, air leakage during cheek puffing, difficulty swallowing, and diminished vocal strength (**Table 2**). Cerebrospinal fluid (CSF) analysis showed no albuminocytologic dissociation in any of the episodes. Myelin basic protein (MBP) was detected in the CSF during the first episode, and serum anti-peripheral nerve antibody profiling during the third episode was positive for anti-sulfatide antibodies (**Table 1**). EMG indicated multiple peripheral nerve injuries in all episodes (**Table 3**). A definitive diagnosis of GBS was established. The patient received two rounds of IVIG (0.4 g/kg/day) for the first episode and one round for the subsequent two episodes resulting in almost complete resolution of symptoms at discharge.

**Table 1:** Clinical features, Hughes scores and CSF findings of three episodes.

	1st	2nd	3rd
Prodromal infection	Respiratory tract infection	Respiratory tract infection	-*
Incubation period (weeks)	2	4	-*
Paralysis of limbs	Yes	Yes	Yes
Sensory disturbance	Yes	Yes	Yes
Cranial Nerve Paralysis	Yes	No	No
Autonomic Dysfunction	Yes	No	No
Mechanical ventilation	Yes	No	No
Time to the nadir of illness (weeks)	3	2	1
Course of disease(weeks)	6	1	1
The Hughes score at the nadir (points)	5	4	3
The Hughes score at discharge (points)	4	2	2
Lumbar puncture distance from onset of illness(days)	14	14	4
CSF protein (g/l)	0.39	0.39	0.46
WBC (s/mm^3^)	5	2	2
Albuminocytologic Dissociation	No	No	No
Special tests	Myelin basic protein(MBP)0.72 mmol/l (normal ≤ 0.55 mmol/l)	-**	Anti-Sulfatide antibody positive
Brain imaging (CT/MRI)	Brain CT scan is normal(4 weeks from onset)	Brain MRI and CT scan are normal (2 weeks from onset)	-**
Other inspection (completed at the time of each admission)	Test for blood routine, liver and kidney function, blood electrolyte panel, coagulation function, D-dimer fibrin levels, serum folic acid, serum Vit B12, thyroid function tests, serological markers for rheumatic and autoimmune diseases, tumor markers, specific infections (including HIV, syphilis and hepatitis B), ECG, echocardiography, abdominal ultrasound, chest CT, and thyroid ultrasound revealed no abnormalities.		

*No history of preceding infection; **Not performed; Hughes Functional Grading Scale (GBS disability scale) (5); CSF, cerebrospinal fluid; CT, Computed Tomography; MRI, Magnetic Resonance Imaging; ECG, electrocardiogram.

**Table 2:** Neurological examinations (Nadir and Discharge) of three episodes.

	1st episode		2nd episode		3rd episode	
	nadir	discharge	nadir	discharge	nadir	discharge
cranial nerve	eye closure weakness, tympanic and cheek air leakage, dysphagia, voice weakness	normal	normal	normal	normal	normal
upper limb muscle strength*	4/5	5/5	5/5	5/5	5/5	5/5
lower limb muscle strength*	2/5	4/5	4/5	5/5	5/5	5/5
muscular tension	normal	normal	normal in both upper limbs, hypotonia in both lower limbs	normal in both upper limbs, hypotonia in both lower limbs	normal	normal
tendon reflex	areflexia	areflexia	hyporeflexia in both upper limbs and areflexia in both lower limbs	hyporeflexia in both upper limbs and areflexia in both lower limbs	hyporeflexia in both upper limbs and areflexia in both lower limbs	hyporeflexia in both upper limbs and areflexia in both lower limbs
superficial sensation	glove (hosiery-like) pinprick pain hypoesthesia in distal extremities	glove (hosiery-like) pinprick pain hypoesthesia in distal extremities	hyperesthesia to pinprick pain and temperature sensation in distal extremities	hyperesthesia to pinprick pain and temperature sensation in distal extremities	hosiery-like pinprick pain hypoesthesia in the distal part of both lower extremities	hosiery-like pinprick pain hypoesthesia in the distal part of both lower extremities
deep sensation	normal	normal	normal	normal	normal	normal
motor coordination	ataxia of both lower limbs	ataxia of both lower limbs	normal	normal	normal	normal

*Using Medical Research Council (MRC) scale (1).

**Table 3:** Results of nerve conduction velocity (NCV) of three episodes.

			1st		2nd		3rd	
			conduction velocity	amplitude	conduction velocity	amplitude	conduction velocity	amplitude
MCV	median nerve	L	normal	normal	normal	normal	normal	normal
		R	–	–	normal	normal	normal	normal
	ulnar nerve	L	normal	normal	normal	normal	–	–
		R	normal	normal	–	–	normal	normal
	popliteal nerve	L	normal	decreased	normal	decreased	normal	decreased with TB
		R	slower	decreased	slower	decreased	slower	decreased with TB
	posterior tibial nerve	L	normal	decreased	normal	decreased with TB	normal	decreased with TB
		R	normal	decreased	normal	decreased with TB	normal	decreased with TB
SCV	median nerve	L	slower	decreased	normal	decreased	normal	decreased
		R	slower	decreased	–	–	normal	decreased
	ulnar nerve	L	not elicited	not elicited	normal	decreased	normal	decreased
		R	not elicited	not elicited	–	–	normal	decreased
	peroneal Nerve	L	normal	decreased	not elicited	not elicited	not elicited	not elicited
		R	normal	decreased	not elicited	not elicited	not elicited	not elicited

MCV, motor nerve conduction velocity; SCV, sensory nerve conduction velocity, “-” means not detected, TB, temporal dispersion.

## 3 Discussion

In this case, the patient experienced three episodes, each reaching its nadir within 4 weeks. The primary symptoms included symmetrical weakness and numbness in the lower limbs, with or without numbness in the fingertips. The first two episodes were preceded by a history of upper respiratory tract infection. Neurological examination revealed hyporeflexia or areflexia in the extremities, and EMG demonstrated multiple peripheral nerve impairments. Based on these findings, the patient meets the diagnostic criteria for GBS. The intervals between recurrences were approximately 5 years and 1 year, respectively, consistent with the diagnostic criteria for recurrent RGBS.

In this case, the patient presented with symmetrical lower extremity weakness, stocking-glove pattern sensory disturbances in distal extremities, cranial nerve involvement, and no neurogenic bladder and bowel dysfunction. The constellation of clinical manifestations, neurological examinations (throughout the course of the disease), and electrophysiological evidence of peripheral neuropathy (demonstrated by electromyography) collectively excluded acute myelitis as a diagnostic consideration. Thyroid function, electrolytes, and thyroid ultrasound results were within normal ranges, excluding the possibility of periodic paralysis and muscle weakness due to hypokalemia. Additionally, tests for folic acid, vitamin B12, rheumatoid immune markers, tumor markers, and specific infections were normal, and there was no history of poisoning. Therefore, nutritional deficiency, tumor related, autoimmune vasculitis, infection related, and toxic peripheral neuropathy were excluded. The nadir of GBS-TRF recurrence typically occurred within approximately 4 weeks, while the intervals between episodes varied, with an average duration of approximately 18 days (6). It is often accompanied by sensory deficits but without distinct electrophysiological abnormalities (7). In this case, the diagnosis of GBS-TRF was excluded as the intervals between recurrences exceeded 2 months, and sensory nerve axonal damage was clearly identified through EMG. Episodes of A-CIDP may evolve into a chronic course lasting more than 8 weeks, with EMG demonstrating persistent deterioration of neurotransmission function and necessitating long-term immunotherapy. In addition, loss of pinprick sensation, cranial nerve involvement, and autonomic dysfunction are rare in A-CIDP (3). In this case, pinprick hyperalgesia, cranial nerve damage, and autonomic dysfunction occurred during the course of the disease. The disease duration was less than 8 weeks in all episodes, symptoms resolved completely following treatment without evolving into a chronic course, and EMG did not indicate significant deterioration of conduction function. Therefore, the diagnosis of A-CIDP can be excluded.

The initial episode was notably severe, involving multiple cranial nerves and culminating in respiratory muscle paralysis, in contrast to the subsequent two recurrences. During the first episode, the patient exhibited weakness in eye closure, puffing out the cheeks with air leakage, dysphagia, hypophonia, respiratory distress, and a sudden increase in heart and respiratory rates. These symptoms were attributed to involvement of the facial nerve, vagus nerve, glossopharyngeal nerve, and hypoglossal nerve resulting in paralysis of the orbicularis oculi and pharyngeal and lingual muscles. Respiratory distress likely resulted from involvement of the phrenic and spinal nerves leading to weakness of respiratory muscles, including the diaphragm, intercostal muscles and abdominal wall muscles. Weakness of these muscles may cause ineffective coughing, hypoventilation, atelectasis, retention of airway secretions, and ultimately respiratory failure (8). Additionally, the patient experienced a sudden increase in heart rate to 126 beats per minute and respiratory rate to 37 breaths per minute at rest. This was attributed to vagal dysfunction, manifesting as sympathetic overactivity leading to increased heart and respiratory rates. Severe autonomic dysfunction occurs in approximately 20% of GBS patients and may result in potentially fatal complications, such as cardiac arrhythmias, severe hypertension, or hypotension (9, 10). Autonomic dysfunction is an independent risk factor in the poor short-term prognosis of GBS patients (11) and is also the most common cause of death in patients with GBS (10), although the exact immunopathologic mechanism remains to be elucidated.

Patients exhibited progressively shorter intervals between relapses consistent with prior reports (2). Notably, Inan B et al. (3) observed that the interval between antecedent infections and symptom onset typically shortens during subsequent relapses accompanied by a moderate reduction in the time from symptom onset to nadir. Most patients reached the nadir within 2 weeks, with nearly all achieving it within 4 weeks (5). While the shortened time to nadir during relapses in our patient aligns with these findings, the prolonged latency between antecedent infections and symptom onset may be linked to variations in pathogen-specific incubation periods or recall bias regarding infection timing. In recurrent RGBS, clinical severity during relapses varies: some patients experience worsening or more severe symptoms, while others exhibit milder or comparable severity relative to the initial episode (2, 3, 12). In this case, progressively lower Hughes functional scores during subsequent relapses indicated reduced symptom severity. This attenuation may reflect either early IVIG administration mitigating symptom progression or a less aggressive secondary immune response targeting peripheral nerve antigens compared to the initial episode. Regarding variations in recurrence intervals, symptom severity, and time to nadir in recurrent RGBS, it is speculated that these may stem from factors such as heterogeneity in study populations, diversity of triggering pathogens, and therapeutic interventions. Whether this pattern is common in RGBS remains inconclusive.

The pathogenesis of GBS is predominantly driven by molecular mimicry, wherein microbial antigens cross-react with peripheral nerve components (e.g., gangliosides) triggering anti-ganglioside antibody production and subsequent complement-mediated nerve injury (10). Approximately two-thirds of GBS cases are preceded by infections, most commonly affecting the respiratory or gastrointestinal tracts (e.g., *Campylobacter jejuni*) (4, 9, 10). Less frequently, non-infectious triggers, such as surgical procedures, trauma, vaccinations, or exogenous ganglioside exposure, have been implicated (3, 4). The mechanisms underlying RGBS remain incompletely elucidated. Notably, antecedent infections during recurrences often differ etiologically from those preceding the initial episode despite comparable clinical phenotypes suggesting that genetic susceptibility and dysregulated host immunity are pivotal to RGBS pathogenesis (2). In this case, the first two episodes followed upper respiratory tract infections, though specific pathogens were undetermined due to the patient’s delayed medical consultation during the prodromal phase. The third episode lacked identifiable triggers; however, admission laboratory studies demonstrated progressive lymphopenia (1.76 × 10^9^/L vs. 1.90 × 10^9^/L in the second episode). Lymphopenia may reflect impaired immune surveillance potentially exacerbating pathogen-induced aberrant immune activation and thereby predisposing to RGBS recurrence. Nevertheless, lymphocyte count holds limited utility as a predictive biomarker due to its inherent biological variability and low diagnostic stability (13).

Albuminocytologic dissociation—characterized by elevated CSF protein levels with normal cell counts—is observed in approximately 80% of GBS patients, though protein levels may remain normal during the initial days of symptom onset (5). In this case, albuminocytologic dissociation was absent, likely due to lumbar puncture timing (performed either before the typical protein elevation window or during the recovery phase when levels normalize). During the first episode, CSF analysis revealed elevated myelin basic protein (MBP) at 0.72 mmol/L (normal ≤0.55 mmol/L). While MBP, a structural component of central nervous system (CNS) myelin, correlates with disease severity in demyelinating disorders, it lacks specificity for GBS, as elevations are also documented in multiple sclerosis (MS) (14). However, MS was excluded in this patient due to normal brain MRI findings and the absence of upper motor neuron signs on neurological examination. A positive serum anti-sulfatide IgG antibody was detected during the third episode (via Western blotting, Tianjin Jinyu Laboratory). A few studies have focused on this antibody. Sulfatide antibody is closely associated with GBS pathogenesis and serves as an important diagnostic biomarker for GBS (15). Although rare in GBS patients, this antibody may specifically occur in GBS cases with widespread sensory loss (16). Sensory axonal peripheral neuropathy correlates with anti-sulfatide antibody deposition on peripheral nerve axons, sensory nerves, and posterior root ganglion neurons (15). GBS patients with severe sensory abnormalities exhibit high antibody positivity rates, with peak titers observed during the acute disease phase and normalization occurring after 3 weeks (16). Yan et al. (15) also reported that anti-sulfatide antibody-positive GBS can present with motor and sensory involvement of the extremities, more pronounced sensory deficits, and neurophysiological evidence of sensory nerve axonal damage; these cases showed good prognoses following immunotherapy. As the clinical symptoms and EMG findings across all three episodes in this patient predominantly indicated sensory nerve damage, along with a clear IVIG treatment response, it is hypothesized that anti-sulfatide antibodies might have been positive during the first two episodes. However, sulfatide antibodies lack specificity for GBS, as elevated titers can also occur in certain neurologic or immunologic disorders (16).

In this patient, EMG findings across all three episodes primarily demonstrated severe axonal damage with concurrent demyelinating features. The demyelinating subtype of GBS is more prone to recurrence compared to the axonal subtype (6). This patient differed electrophysiologically from pure sensory GBS patients who typically exhibit predominantly demyelinating sensory nerve changes. This distinction may reflect that sensory axonal damage may be different in patients with anti-sulfatide antibody-positive GBS (15).

Yuki N et al. (9) showed that for patients with severe and refractory symptoms, a second course of IVIG is beneficial. During the patient’s first episode, two courses of IVIG were administered. In the first episode, the symptoms continued to progress after the first course of IVIG treatment culminating in respiratory distress that required tracheal intubation and mechanical ventilation. Due to difficulty in weaning from the ventilator, a second round of IVIG was initiated. Following two courses of IVIG, the patient exhibited significant improvement in respiratory muscle function and bilateral lower limb strength. The subsequent two recurrences were each treated with a single course of IVIG resulting in favorable recovery of symptoms and clinical signs. It is noteworthy that patients with RGBS typically do not require long-term immunotherapy (3). However, patients with GBS-TRF often benefit from repeated IVIG or plasma exchange (PE), while those with A-CIDP usually require long-term maintenance therapy with corticosteroids, IVIG, or PE (7).

At the 1-month post-discharge telephone follow-up, we confirmed persistent residual numbness localized to the right foot. While we recommended repeat EMG testing to evaluate neurophysiological progression, the patient declined further EMG evaluation, thereby precluding objective documentation of neurological improvement. While GBS generally has a favorable prognosis, some cases still result in mortality or severe disability. The prognosis for this patient remains challenging to evaluate: although axonal GBS is associated with poorer outcomes (10), anti-sulfatide antibody-positive GBS patients typically exhibit favorable prognoses (15). This case report aims to enhance neurologists’ recognition of RGBS.

## Data Availability

The original contributions presented in the study are included in the article/Supplementary Material. Further inquiries can be directed to the corresponding author.
